# Too Important to Ignore: Leveraging Digital Technology to Improve Chronic Illness Management Among Black Men

**DOI:** 10.2196/jmir.9434

**Published:** 2018-05-14

**Authors:** Stuart W Grande, Ledric D Sherman

**Affiliations:** ^1^ The Geisel School of Medicine at Dartmouth The Dartmouth Institute for Health Policy and Clinical Practice Dartmouth College Hanover, NH United States; ^2^ College of Education and Human Development Department of Health and Kinesiology Texas A&M University College Station, TX United States

**Keywords:** black men, digital health, chronic illness

## Abstract

Health disparities associated with chronic illness experiences of black men demonstrate widespread, systematic failures to meet an urgent need. Well-established social and behavioral determinants that have led to health disparities among black men include racism, discrimination, and stress. While advocacy work that includes community-engagement and tailoring health promotion strategies have shown local impact, evidence shows the gaps are increasing. We suspect that failure to reduce current disparities may be due to conventional public health interventions and programs; therefore, we submit that innovative interventions, ones that embrace digital technologies and their ability to harness naturally occurring social networks within groups, like black men, have particular importance and deserve attention. This commentary characterizes the current literature on chronic illness among black men as well as health interventions that use digital technology, to build a case for expanding research in this area to reduce the overwhelming burden of chronic illness among black men.

## Prioritizing Chronic Illness Management Among Black Men

There is no shortage of data on the debilitating effects of health disparities, particularly on the effects of chronic illness among black men. If these data are so compelling, then why hasn’t the story changed? Many have argued this is a moral issue that is centered on the principle that suffering due to race, gender, or genetics is unconscionable. Despite how often these injustices are professed or predicted, these moral commitments and righteous indignations fall short. In this commentary, we take the position that failures to reduce health disparities among black men are the result of more traditional community-engagement approaches that reflect passive education and one-off health promotion activities. We apply the term black men inclusively to refer to any Hispanic or non-Hispanic man who may or may not self-identify as African American. Further, borrowing from the world of health services research, we contend that patient engagement strategies—those promoted by digital technology and where the key ingredient is partnership and collaboration—offer promising advances to mitigate health disparities experienced by black men [1,2]. Simply put, the mounting evidence on the potential of these technologies is too great to ignore. An innovation of this caliber is necessary to refocus attention to enduring gaps in health service delivery systems and to support black men in ways that are the most meaningful to them. In this regard, digital innovation must emerge from foundations of partnership and collaboration, where preferences and needs drive technology that guides self-management of chronic illness.

One of the leading contributors to morbidity among black males is chronic illness, particularly heart disease and high rates of hypertension (about 43% of black men have hypertension compared with about 30% of white men) [3]. The chronic illness burden among black men in the United States is disproportionate compared to other male ethnic and racial groups [4]. Unmanaged chronic illness, where illness symptoms go untreated and advance in severity, also adversely impact the health of black men. According to the Centers for Disease Control and Prevention (CDC), 7 of the top 10 causes of death among black men in 2014 were related to chronic illness, two of which—heart disease and cancer—comprise nearly 46% of all deaths [5]. As unmanaged chronic illness occurs based on several factors, we suspect that black men are more often unsupported to navigate the healthcare system in a way that makes sense for them. We are aware that black men face a wide set of systematic barriers, mostly out of their control, related to accessing and engaging in supportive and beneficial health promotion activities. This suggests that alternative approaches are necessary.

Since the publication of *Unequal Treatment in 2003*, the causal pathways of health disparities are well known, and yet, insufficient change has occurred. Over the last several years in the United States, while access and quality have improved, health disparities remain consistent across groups [6]. A recent review of black male health examines how the healthcare system affects their lived experiences, making the point that social and behavioral determinants that would positively impact their lives are under-supported and neglected [7]. There is a trend of decreasing life expectancy in black vs whites; and among men, this trend remains persistent. One of the most credible factors is higher rates of uncontrolled hypertension or high-blood pressure, often reflected as an individual with high blood pressure not receiving treatment. While control of hypertension is linked to higher quality care, there are reports that better communication strategies with doctors are vital, especially for developing successful treatment strategies [8]. Knowing that discrimination, racism, and stigma negatively impact black men and their engagement with the healthcare system, building communication strategies that counteract these biases is critical [9]. We take the view here that the goal of overcoming barriers to engagement must be the centerpiece of novel approaches. These approaches may not be practical at the system-level but may be possible at the individual level. Therefore, designing solutions at the individual level so that systems can better support how black men engage in healthcare may have the highest likelihood of success.

Much of the current literature on black men and health reflect outcomes associated with poverty, education, discrimination (racism), and poorly managed stress [7]. It is unreasonable to think that these can be attenuated by health education or one-off health promotion events alone. Understandably, these experiences are highly personal and further complicated by chronic illness management, which requires informed medical support. Scholars and practitioners alike see little benefit to simply identifying these disparities and failing to build platforms aimed toward changing current systems. While community-based participatory methods have emerged as a leading strategy for engaging racial and ethnic groups, fostering empowerment through trust building and transparency remains a major hurdle [10]. Given these gaps and opportunities, might building digital technologies that are designed to meaningfully engage black men, prioritize the contribution of brotherhood and existing social networks among black men [11]? Here we adopt Franklin’s definition of brotherhood as a “community of African American or black men connected through tacit understanding, common experiences, traditions, and identity.” It is this level of connection that has been until recently, mostly unexplored, and offers exciting areas co-designing interventions to reduce disparities [12].

Integrating health interventions within trusted social systems like a brotherhood, built around shared beliefs, attitudes, and authentic relationships may overcome many recognized barriers of current strategies [13]. To this end, we argue that digital technologies present a unique potential for building meaningful solutions to current challenges associated with managing chronic illness among black men.

## Technology as a Means of Engagement

Mobile digital devices and apps, including websites and social media platforms, offer immediate access to medical and health information on the internet and also offer new ways of monitoring, measuring, and visualizing the human body while sharing personal information and experiences with others [14]. Electronic or digital health encompasses a wide range of technologies that are used for healthcare, health informatics, health education, and health promotion. Mobile health (mHealth)—the application of wireless technology to promote wellness and information provision between healthcare and individuals—is rapidly gaining attention in both preventive medicine and chronic illness management [15]. One of mHealth’s strengths is its ability to leverage the existing mobile technology infrastructure and the ubiquity of the mobile phone across populations and subgroups. Previous research has corroborated the high prevalence of smartphone usage among blacks as well as Hispanics and indicated the value of mHealth in managing chronic illness [14].

Smartphone and tablet apps can educate, assist with decision-making, and promote adherence to lifestyle and medication regimens. Consumer-wearable technology comprises devices equipped with sensors and wireless connectivity that can assist with a wide range of health-related applications such as monitoring blood sugar levels, personalizing treatment, connecting with health-care providers, and even delivering medication into the body [15]. Recent work in diabetes and obesity shows technological advancement in the use of wrist and hip accelerometers, global positioning system (GPS), mobile technology apps for weight loss, apps for dietary self-monitoring, internet weight loss programs, and behavioral intervention technologies. While there is some fear regarding information security and potential Health Insurance Portability and Accountability Act (HIPAA) concerns, potentially opt-in rather than opt-out features offer individuals needed flexibility to assume or reject risk based on preference [16].

## New Opportunities with Digital Technologies

Developments in new technologies go beyond self-monitoring. Some include prevention of illness complications such as diabetic neuropathy, and others, like prototype socks and shoes with thermal and pressure sensors, can identify areas of the feet with insufficient blood supply [17,18]. Potentially, a physician could use this type of technology to routinely inspect minor tissue damage and greatly minimize the risk of amputations. In the more immediate future, technologies like these will offer practical approaches to managing chronic illnesses like diabetes. *The OneTouch Reveal* mobile app, for example, can test a drop of blood and tell whether sugar levels are within normal range as well as provide a summary of overall health performance [19]. Another app, called *Diabetik,* provides quick and interactive data entry to monitor diet, blood glucose levels, and medication [20]. The user can set medication or activity reminders according to time or location. *Fooducate* has an extensive database of food information that uses barcode scanning to search nutritional value and suggest healthier alternatives [21]. Uniquely, this app also creates a community in which users can share progress and healthy recipes.

Although fewer in number, apps that meaningfully connect patients to doctors, like *Glooko*, that aggregates biometric data-glucose monitoring and fitness information - offer physicians novel ways to engage with vital patient information [22]. These data are critical and often overlooked during face-to-face consultations. By presenting this data in easy-to-manage formats, these types of apps support patient and physician engagement that contribute to robust dialogue and more efficient clinical visits. These apps also provide the opportunity to foster new conversations that both patients and doctors may have been too intimidated or preoccupied to engage.

Weight management apps are available that focus primarily on tracking and require a certain amount of user-input to manage. These act more like calendars and reminders to record the food and drink intake. Apps also let users track your exercise using information that you enter manually or by tracking your activity automatically using the GPS or accelerometer in your phone. Compared to using paper diaries, tracking via smartphone may be more convenient because the device does some of the work for you (eg, calories, movement tabulations), but these are only for self-monitoring purposes. These and other wearable devices typically display the information collected and provide feedback via a smartphone app and website, allowing you to track changes over time. Importantly, one trial showed strong evidence for the use of smartphone technology for managing weight, giving us hope for broader interventions in communities where managing chronic illness are a challenge [23].

## Tailoring Digital Technology for Black Men

Tailoring interventions for black men makes good sense given existing communication networks within subgroups. Digital technology is uniquely positioned to attend to subgroup differences as they are, by nature, personal and self-managed. Others point to the importance of system-level identification and intervention using structural factors, but these, while necessary, are too general and are less accessible than digital technology [24]. To date, there is good evidence for targeted and tailored interventions in black communities [25]. In this sense, a tailored intervention would be more appropriate than simply modifying language or applying a “one size fits all” approach to effectively meet black men where they are. According to Hawkins and colleagues, this would include personalization, gaining feedback from black men, and matching content to their personal data [26]. As identified elsewhere, community-based participatory research (CBPR) strategies are effective evidence-based methods for engaging, co-designing and tailoring mHealth interventions to meet preference-driven solutions [27].

Despite the limited amount of published mHealth interventions to improve chronic illness among black men, the existing research on computer-tailored and social media interventions offer some insight. Consensus seems to suggest that tailored applications should be: theory-based, contextual, modifiable to individual preferences, able to connect black men to other black men, include a preference-based dashboard, and be supported by popular culture references [28]. Informed by CBPR, mHealth interventions must be developed and optimized for black men. Taken a step further, the perspective supports a more intersectional approach to building more impactful and effective interventions. Research that targets the intersection of social, health, context, and identity overcomes the myopic view of black men’s health. This complex and nuanced viewpoint challenges this view by suggesting that an app that mediates naturally occurring social networks of black men with type 2 diabetes will consider the overlap of both gender and the marginalization of race [29].

Further, we see that tailoring can and should mean developing interventions (in this case mHealth apps) in partnership with black men. For example, if developers choose a deep tailoring approach rather than a surface tailoring one, the mHealth apps will integrate cultural values, norms, and religious or spiritual beliefs of the population of interest (black males) [30]. Examples of these tailoring messages might include age-appropriate material to engage churches, ministry teams, and fellowship groups in the development and integratration of popular culture messaging/references. In addition, using content specific (chronic illness) framing along with connectivity platforms, enhances relationship building and help seeking around chronic illness management [31]. Such tailoring would also support the existing social networks and relationships aligned with brotherhood, as described earlier.

There is also thinking that bias and preference play a role in the perpetuation of race-based health disparities, but often these are superseded by communication challenges [32]. The systematic bias in health delivery and its contribution to a perpetuation of fear and apprehension among black men is widely recognized [24]. As shown, attending to individual fears about talking with physicians is necessary for improving confidence and readiness (activation) for engaging in open and honest dialog [33]. And while the bias issue may overstate the role of the clinician in this relationship, an argument can be made that provided the right support platform, black men offer each other the best strategies.

Health services research has demonstrated that individuals who feel more empowered and confident to make decisions are healthier [34]. These broad approaches have shown efficacy in other areas, but when it comes to tailoring interventions for black men, there is a need to leverage platforms for social support. By providing access to health resources, important health information that relies on and utilizes recognized social support can be more effective particularly with self-management and adherence [35]. This strengthens the argument that innovative technologies designed to leverage social ties have the greatest likelihood of success. The challenge will be determining best practices. What is needed is efficient processes and communication to improve self-efficacy, the confidence to participate meaningfully in one’s health.

## Feasibility of Digital Technology for Advancing Black Men’s Health

There have been many efforts to mitigate disparities among black men. Given the scope and trends of current disparities, we suggest that technology, and particularly digital technology focused on engagement and activation, can address some of these gaps.

There is currently a wide gap in exploring the use of these technologies among black men to empower and encourage health behaviors and self-management strategies. In 2000, a survey of internet usage among blacks showed they were 38% more likely than whites to seek information about jobs on the web, and 45% of African-Americans vs 35% of whites say that the internet helps them find health care information [36]. A 2018 Pew fact sheet shows that 98% of blacks own a cell phone, and 75% own a smartphone, suggesting that opportunities to download and utilize online content are wide and far-reaching [37]. We also see that young African-Americans have high use of Twitter, and 96% of those aged 18–29 use some social networking platform [38]. The high frequency of socializing using these new technologies supports our efforts to capitalize on social norms in the community.

There are multiple initiatives that support person–centered design, and tailoring of messaging in ways that have been minimally examined among black men. For example, health and medical support and information on websites, apps, and social media sites have proliferated, facilitating the access of lay people to health-related information and providing them with the opportunity to share experiences of their illnesses or health-promoting activities. Health promotion researchers have sought to investigate how websites and social media sites operate in generating and disseminating information about strategies for promoting health among lay people who do this voluntarily as part of social interactions and support systems. Further, social support systems that foster pre-existing social networks, characteristic of the brotherhood as defined earlier have been unexplored and offer exciting areas for mitigating disparities [12].

## Embracing Novel Technologies to Support Engagement and Chronic Illness Management

The health disparities that describe the population health outcomes for black men in the United States has garnered much attention, and yet the story remains the same. Failure to reduce current disparities may be due to conventional public health interventions; therefore, we submit that innovative interventions, ones that embrace digital technologies and their ability to harness inherent social trends within groups, like black men, have particular importance and deserve attention. Further, we outline alternative interventions to mitigate chronic illness in black men as a way to break the dominance of current deficit-based approaches that frame behaviors as negative or abnormal.

We recognize that medical anthropologists have also weighed in on the debate, suggesting that technologies that seemingly enhance the patient experience may also foster a false sense of hope—what Mary-Jo DelVecchio Good refers to as the “medical imaginary” [39]. The hope of technology seems to be conflated in concepts of innovation and improvement, where hope sits more with consumers than providers. This debate on the benefits of user-centered technologies for patient outcomes seems much less controversial when we reflect on non-hospital based approaches. In other words, the potential of these technologies seems most impactful in communities, where engaging individuals through tailored approaches have the most likely benefit.

According to Forbes one of the top five most important innovations in health care is the use of digital technology for empowering consumers [40]. While many have suggested that black men are members of hard-to-reach populations, with a history of not participating in much research, there is literature debunking these myths. In fact, when researchers make an effort to engage black men in meaningful ways, the evidence is strong that positive behavior change is not only possible but dynamic. What is needed are interventions that show promise across other groups, based on co-design strategies that prove to engage and empower black men to make healthy choices around the management of their chronic illness. We believe that if designed properly these technologies offer needed ways to more fully connect communities to health care resources and professionals. We have hopefully built a case for expanding research in this area to reduce the overwhelming burden of chronic illness among black men.

## Abbreviations

CBPR: Community-Based Participatory Research
CDC: Centers for Disease Control and Prevention
GPS: global positioning system
HIPAA: Health Insurance Portability and Accountability Act
mHealth: mobile health
